# Paleovegetation Community and Paleoclimate Succession in Middle Jurassic Coal Seams in Eastern Coalfields in Dzungaria Basin, China

**DOI:** 10.3390/plants14050695

**Published:** 2025-02-24

**Authors:** Xingli Wang, Shuo Feng, Wenfeng Wang, Qin Zhang, Jijun Tian, Changcheng Han, Meng Wang

**Affiliations:** 1 Xinjiang Key Laboratory for Geodynamic Processes and Metallogenic Prognosis of the Central Asian Orogenic Belt, Xinjiang University, Urumqi 830047, China; 18797225936@163.com (X.W.); wenfwang@163.com (W.W.); 107552304890@stu.edu.cn (Q.Z.); hanchangcheng@xju.edu.cn (C.H.); 2 School of Resources and Geosciences, China University of Mining and Technology, Xuzhou 221116, China; wangm@cumt.edu.cn; 3 School of Earth Resources, China University of Geosciences, Wuhan 430074, China

**Keywords:** Dzungaria Basin, paleoclimate, paleovegetation, paleovegetation community, spores and pollen

## Abstract

The Dzungaria Basin is located north of Xinjiang and is one of the largest inland basins in China. The eastern coalfields in the Dzungaria Basin contain a large amount of coal resources, and the thickness of the coal seams is significant. Therefore, the aim of this study was to classify the paleovegetation types and develop paleoclimate succession models of the extra-thick coal seams. We conducted the sampling, separation, and extraction of spores and pollen and carried out microscopic observations in the Wucaiwan mining area of the eastern coalfields in the Dzungaria Basin. The vertical vegetation succession in the thick seam (Aalenian Stage) in the study area was divided into three zones using the CONISS clustering method. The results show that the types of spore and pollen fossils belong to twenty families and forty-five genera, including twenty-three fern, twenty gymnosperm, and two bryophyte genera. The types of paleovegetation in the study area were mainly Lycopodiaceae and Selaginellaceae herb plants, Cyatheaceae, Osmundaceae, and Polypodiaceae shrub plants, and Cycadaceae and Pinaceae coniferous broad-leaved trees. The paleoclimate changed from warm–humid to humid–semi-humid and, finally, to the semi-humid–semi-dry type, all within a tropical–subtropical climate zone. The study area was divided into four paleovegetation communities: the nearshore wetland paleovegetation community, lowland cycad and Filicinae plant community, slope broad-leaved and coniferous plant mixed community, and highland coniferous tree community. This indicates that there was a climate warming event during the Middle Jurassic, which led to a large-scale lake transgression and regression in the basin. This resulted in the transfer of the coal-accumulating center from the west and southwest to the central part of the eastern coalfields in the Dzungaria Basin.

## 1. Introduction

Currently, paleontological data on spores and pollen are commonly used for paleoenvironmental and paleoecological reconstruction. A detailed study of the types and quantities of spores and pollen from various continuous strata can help deduce the paleoenvironment [1,2]. Vera A. Korasidis et al. examined a model combining the spores and pollen of various coal seams in the Latrobe Valley, Gippsland Basin, Australia. They summarized the distribution features of the paleovegetation and determined the characteristics of the paleoclimate and paleoenvironment during various depositional periods [3,4]. The coal petrography characteristics of the Cambay Basin, Makum Coalfield, and Rajasthan state showed that the ecological plant community was mixed [5,6]. The variety of spore and pollen combinations in the vertical direction ultimately determines the types of paleovegetation in the original coal formation. In China, spore and pollen combinations are also commonly used to reconstruct the paleoenvironment. The Yangye Formation is located in East Subashi of the western Kunlun Mountains and was formed in the early Middle Jurassic period. Its depositional stage was confirmed through the study of its spores and pollen [7]. The study indicated a warm and humid paleoenvironment with a warm–temperate or subtropical paleoclimate [8,9]. The research on the paleovegetation fossils, spores, and pollen in the Dananhu and Shaerhu Coalfields in northern Xinjiang has shown that the paleovegetation mainly consisted of Filicinae and cycads [10,11,12,13]. The deposition of the extra-thick coal seams in the Middle Jurassic period likely occurred under very high temperatures.

The eastern coalfields in the Dzungaria Basin (ECDBs) contain significant coal resources, with proven cumulative coal reserves of approximately 231.6 billion tons, making them one of the largest coalfields in China. Previous studies have mainly focused on the division and comparison of the coal-bearing strata; research on the formation of single extra-thick coal seams and the succession of paleovegetation has seldom been conducted. Although previous research on Early Jurassic and Late Jurassic sporopollen work in the Dzungaria Basin has been relatively thorough, it has mainly concentrated on the southern margin, northwest margin, and central and western regions of the basin; research on the classification of vegetation types and paleoclimate through sporopollen analysis in the eastern part of the basin is relatively limited. In this study, 44 coal samples were collected from 22 coal seam sequences in a typical section of the Wucaiwan mining area in the east of the Dzungaria Basin. Through separation and extraction experiments, we identified sporomorphs, found 45 genera of 20 families, and analyzed their proportions and change trends. Coal-forming plant types and paleoclimate succession laws in the study area were also studied, thus providing evidence for the identification of coal-forming materials in extra-thick coal seams and a scientific basis for the evaluation of coal-forming paleoenvironments.

## 2. Geological Setting

Dzungaria Basin is located in northern Xinjiang at the junction of the Altai Mountains and Tianshan Mountains. It is bordered by the Dzungaria western mountainous region to the west and the Baytik Piedmont to the east [14]. The shape of Dzungaria Basin is an irregular triangle, with the northern part at a higher altitude than the southern part. Influenced by east–west compression stress, Dzungaria Basin has developed a series of contemporaneous sedimentary faults. The sag and salient structures trend north–east or north–south. The sag structures led to the formation of coal-bearing strata, while the salient structures did not [15,16]. The basin is rich in coal, petroleum, and gas resources. The main target for this study was the coal seam of the Aalenian Stage from the Middle Jurassic period in the Wucaiwan depression located in the eastern Dzungaria Basin [17]. The thickness of the seams in the research area is 63 m. The coal has a low ash content, low sulfur content, and high caloric value, presenting high commercial value due to the proven substantial coal reserves.

**Figure 1:**
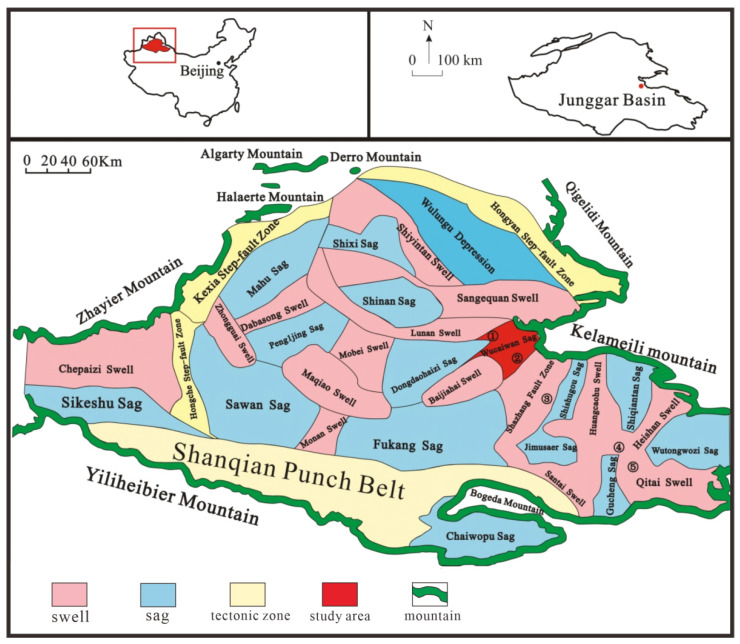
Geological map of Dzungaria Basin. ① Lucaogou exploration area; ② Wucaiwan exploration area; ③ Dajing exploration area; ④ Xiheishan exploration area; ⑤ Jijihuxi exploration area (modified from [30]).

During the Indo-China Movement, the orogenic zones around the research area experienced further uplift and developed thrust nappe structures due to the proximity of the Qinling–Kunlun Ocean and the rise of the Kunlun–Qinling belt in the Late Triassic period [18,19,20]. Coal-bearing formation was minimal because of the overall uplift of the basin and mountains [21,22,23]. During the early Yanshanian period, the uplift of the basin and mountains became stable, leading to the development of broad basins and large lakes in the Early and Middle Jurassic periods, which culminated in the largest coal accumulation event [22,24,25,26,27]. During the middle and late periods of the Yanshan Movement, the reactivity and uplift of the orogenic zone in the northwest increased due to the extrusion stress from both the East Asia multi-directional convergent tectonic system in the north and the closure of the middle Tethys Ocean Basin in the south. This led to the disintegration of the large basin and the shrinkage of lake basins, accelerating the development of the reverse faults and halting the coal-bearing formation [14,17,28]. In the Aalenian Stage, the provenance mainly originated from the Kelameili Mountains in the north. The coal seams had typical district development with various coal enrichment centers. Four independent depressions were formed due to the influence of the Indo-China and Yanshan Movements: the Wucaiwan depression, the Shishugou depression, the Shiqiantan depression, and the Wutongwozi depression [29]. These depressions formed relatively independent artesian basins due to the differences in sedimentation. This study was performed in the Wucaiwan depression (Figure 1).

## 3. Materials and Methods

**Figure 2:**
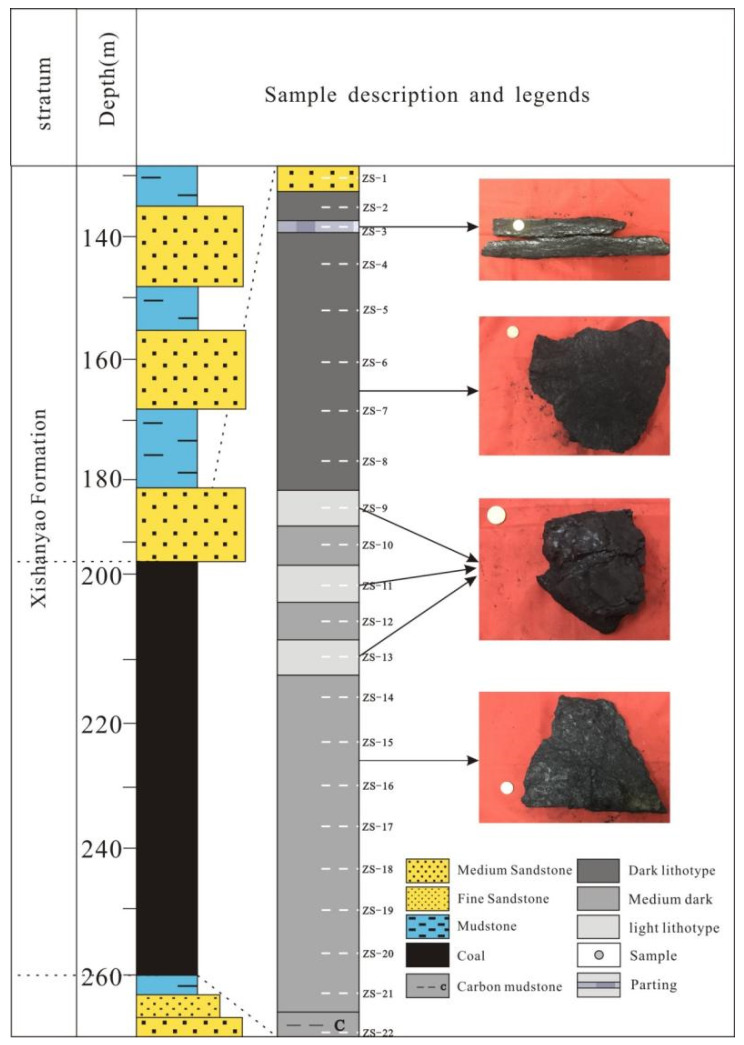
Lithology of the borehole with sample details.

A total of 44 coal samples were collected from 22 coal seam sequences in the Wucaiwan coal mine in the ECDBs based on the *Sampling of coal seams* (GB/T 482-2008, National Standard of the People’s Republic of China) [31]. The analysis and identification of spores and pollen followed the Petroleum and Natural Gas Industry Standards of the People’s Republic of China (SY/T 5915-2000) [32] (Figure 2).

First, the coal samples were crushed to grain sizes ranging from 0.6 to 1 mm. Second, the samples were treated with three different acids: 30% HCl for 24 h, 40% HF for 4 days, and HNO_3_ for 6 h. These treatments removed the carbon, siliceous material, and some of the organic matter from the coal. Then, fresh water was added until the solution was neutral. The solution was then centrifugated at 2000 rpm for 5 min. Subsequently, the solution was discarded, and a new heavy liquid with a density of 2.0–2.2 g/cm^3^ was added. The coal samples were centrifuged at 2000 rpm for 10–30 min. Next, the coal samples were placed in fresh water for another 2 h. Finally, the spores and pollen were filtered with a screen mesh, dipped in glycerogelatin, placed on the center of a slide glass, heated, and covered with a cover glass for further preservation.

In order to visualize the contents of spores and pollen, we used Tilia v.1.7.16 software (Grimm, 2011 [33]) to draw charts based on their relative abundances and then used its built-in clustering analysis (constrained incremental sum of squares, CONISS) for regional division (Grimm, 2011 [33]). According to the CONNIS clustering analysis, three regions were identified and named A, B, and C.

The spores and pollen were observed and photographed under an Axio Scope A1 microscope (Zeiss, Jena, Germany). The identification of sporomorphs was based on The China Sporomorph (Volume II): Mesozoic Spores and Pollen [34], with additional references to the classic literature and consultations with renowned palynology experts.

## 4. Results

### 4.1. Species Composition

A total of 22 spore and pollen samples contained abundant sporomorphs (>150 grains per slide glass). The types of spores and pollen belonged to 20 families and 45 genera. Among them, ferns represented eleven families and twenty-three genera, gymnosperms represented eight families and twenty genera, and bryophytes represented one family and two genera [35] (Table 1).

The samples included the following:

Fontinalaceae: Polycingulatisporites and Sphagnumsporites;

Lycopodiaceae–Selaginellaceae: Aratrisporites, Lycopodiumsporites, Densoisporites, and Neoraistrickia;

Equisetum: Hymenophyllumsporites and Lophotriletes;

Sphenopsida: Calamospora;

Osmundaceae: Osmundacidites, Cyclogranisporites, Baculatisporites, Punctatisporites, and Todisporites;

Cyatheaceae: Cyathidites and Deltoidospora;

Dicksoniaceae: Cibotiumspora;

Dipteridaceae: Dictyophyllidites and Concavisporites;

Gleicheniaceae: Gleicheniidites;

Lygodiaceae: Cicatricosisporites and Klukisporites;

Ophioglossaceae: Undulatisporites;

Polypodiaceae: Polypodiisporites and Laevigatosporites;

Pteridospermae: Caytonipollenites and Alisporites;

Cycadaceae: Eucommiidites, Monosulcites, and Cycadopites–Chasmatosporites;

Araucariaceae: Araucariacites;

Podocarpaceae: Podocarpidites;

Cupressaceae: Perinopollenites;

Cheirolepidiaceae: Classopollis;

Pinaceae: Pinuspollenites, Cedripites, Pseudopicea, Laricoidites, Protopinus, Rugulatisporites, and Piceaepollenites;

Pinaceae, unknown classification: Pristinuspollenites, Rugubivesiculites, Quadraeculina, and Cerebropollenites.

### 4.2. Combination of Spores and Pollen

**Figure 3:**
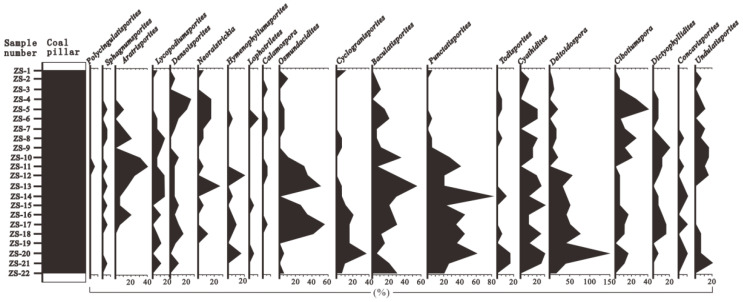
Spore and pollen diagram showing percentage values of main taxa in samples extracted from the extra-thick coal seams (continued in Figure 4).

**Figure 4:**
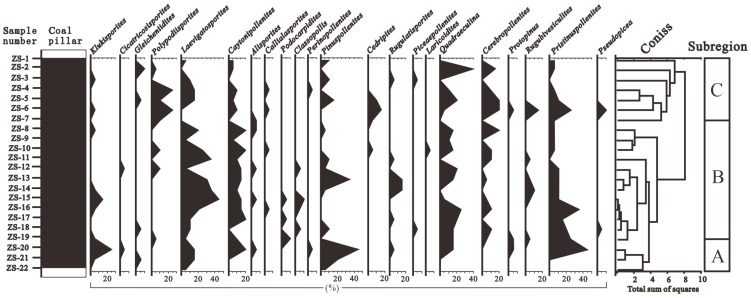
Spore and pollen diagram showing percentage values of main taxa in samples extracted from the extra-thick coal seams (continued from Figure 3).

Based on the changes in the vertical abundance of pollen, and using the CONISS algorithm of the Tilia software to cluster and analyze the pollen data, the thick coal seams in the Wucaiwan mining area of Zhundong were divided into three zones from the bottom to the top: A, B, and C (Figure 3 and Figure 4; [33]).

#### 4.2.1. Group A

The sedimentary thickness of Group A was 9 m, which included three spore and pollen samples, labeled ZS-20 to ZS-22. Group A contained eighteen fern, eight gymnosperm, and one bryophyte genera.

**Figure 5:**
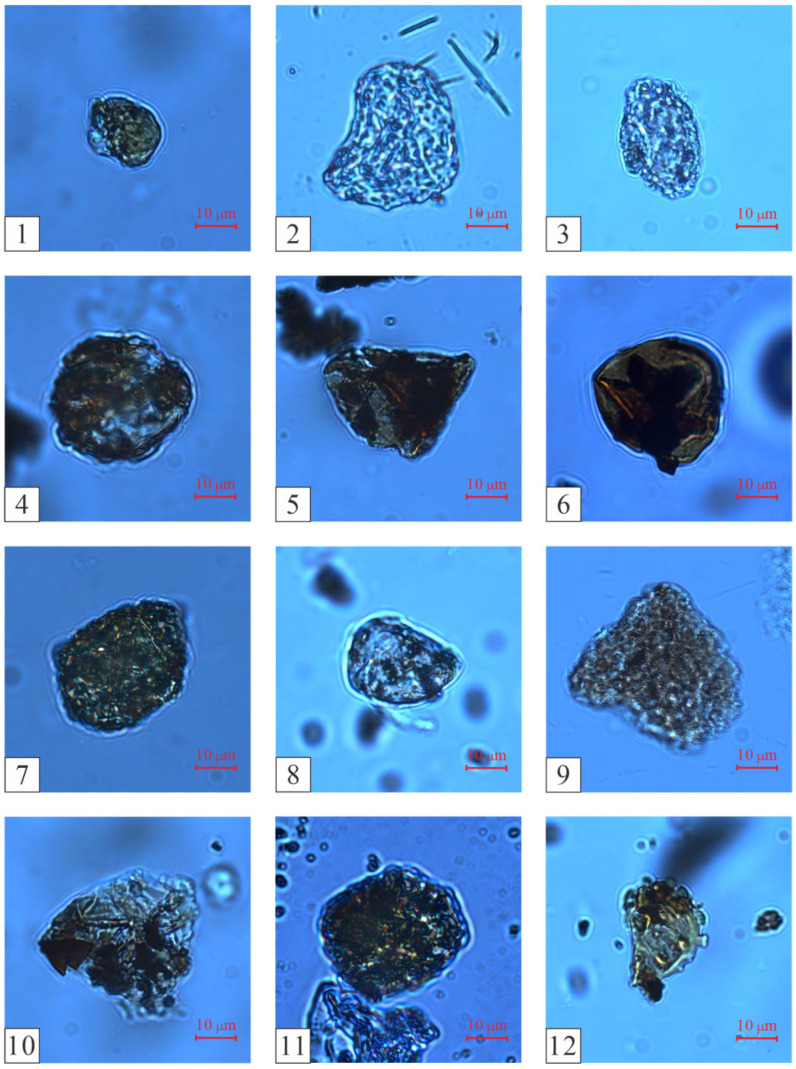
Spore and pollen plate photographs from the extra-thick coal seams of the Aalenian Stage in the ECDBs (Part 1). (1) *Baculatisporites*, (2) *Osmundacidites*, (3) *Osmundacidites*, (4) *Osmundacidites*, (5) *Cyathidites*, (6) *Cyathidites*, (7) *Lycopodiacidites*, (8) *Lycopodiacidites*, (9) *Lycopodiacidites*, (10) *Lycopodiacidites*, (11) *Lycopodiacidites*, and (12) *Lycopodiacidites*.

**Figure 6:**
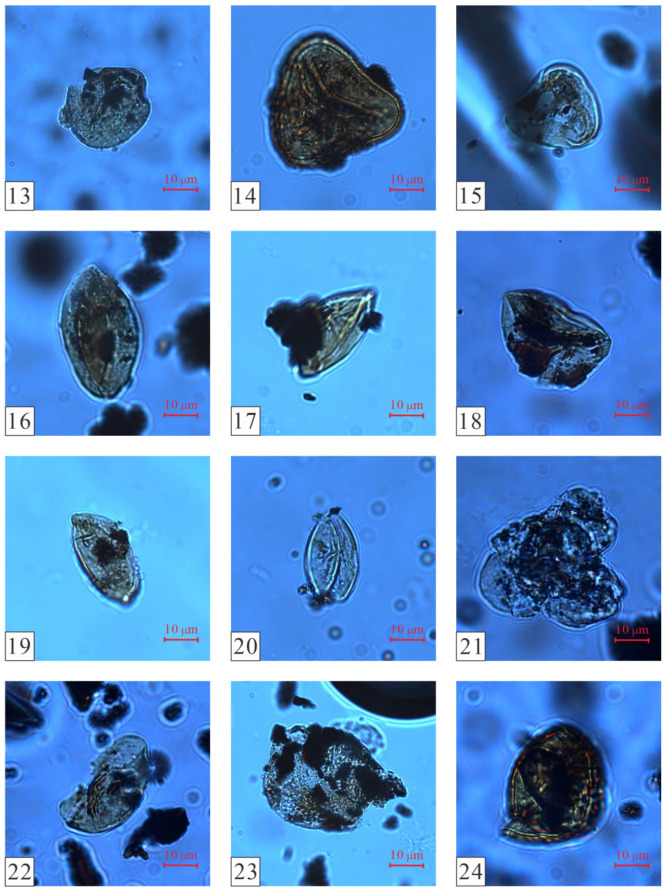
Spore and pollen plate photographs from the extra-thick coal seams of the Aalenian Stage in the ECDBs (Part 2). (13) *Lophotriletes*, (14) *Dictyophyllidites*, (15) *Dictyophyllidites*, (16) *Cycadopites*, (17) *Cycadopites*, (18) *Cycadopites*, (19) *Cycadopites*, (20) *Chasmatosporites*, (21) *Pinuspollenites*, (22) *Pinuspollenites*, (23) *Cerebropollenites*, and (24) *Classopollis*.

The proportion of fern plants ranged from 26.67% to 49.43%, averaging 40.61%. The dominant species were *Osmundaceae* (Figure 5(1–4)) and *Cyatheaceae* (Figure 5(5,6)), ranging from 8.48 to 34.29% and from 7.88 to 20.11%, respectively, followed by *Polypodiaceae* (1.72–2.86%) plants. Other ferns had relatively low contents and no obvious changes, for example, *Lycopodiaceae–Selaginellaceae* (Figure 5(7–12)), *Sphenopsida* (Figure 6(13)), *Dipteridaceae* (Figure 6(14,15)), and *Dicksoniaceae*. Gymnosperms occupied a proportion ranging from 11.43% to 34.5%, with an average of 19.96%. *Cycadaceae* (Figure 6(16–20)) were the dominant species, ranging from 8.57% to 20.12%, followed by *Pinaceae* (Figure 6(21,22)) and *unclassified Pinaceae* plants (Figure 6(23)), with proportions ranging from 2.42% to 6.33% and from 0% to 6.89%, respectively (Table 1).

#### 4.2.2. Group B

The sedimentary thickness of Group B was 36 m, which included 12 spore and pollen samples, labeled ZS-8 to ZS-19. Group B contained twenty-three fern, nineteen gymnosperm, and two bryophyte genera. Compared with Group A, Group B was more abundant in paleovegetation types.

The ratio of ferns ranged from 38.78% to 57.13% (average of 50.21%). The dominant species included *Lycopodiaceae–Selaginellaceae* (3.06–12.22%), *Osmundaceae* (4.80–27.55%), and *Cyatheaceae* (0–16.52%) plants, followed by *Dipteridaceae*, *Polypodiaceae*, *Ophioglossaceae*, and *Hymenophyllaceae*. Group B also contained a minor proportion of *Lygodiaceae* plants and *Gleicheniaceae*. The gymnosperms occupied a proportion ranging from 13.96% to 43.91%, with a slight increase and an average of 26.48%. The *Cycadaceae* (6.96–24.10%), *unclassified Pinaceae* (3.33–19.69%), and *Pinaceae* (0–8.70%) plants were the dominant species among the gymnosperms. *Cheirolepidiaceae* (Figure 6(24)) and *Podocarpaceae* plants were also present. The number of bryophytes ranged from 0% to 2.22%, with an average of 0.51%; *Fontinalaceae* plants were the dominant species (Table 1).

#### 4.2.3. Group C

The sedimentary thickness of Group C was 18 m, which included seven spore and pollen samples, labeled ZS-1 to ZS-7. Group C contained twenty-one fern, seventeen gymnosperm, and one bryophyte genera.

The number of ferns varied from 30.44% to 63.41% (average 42.26%). The dominant species were *Osmundaceae* (2.44–13.04%), *Lycopodiaceae–Selaginellaceae* (0–19.51%), *Cyatheaceae* (0–10.72%), and *Polypodiaceae* (3.33–19.51%). The gymnosperms occupied a proportion ranging from 26.84% to 54%, with an average of 43.84%. The *Cycadaceae* (9.76–37.49%), *Pinaceae* (0–14.29%), and *unclassified Pinaceae* (0–40%) plants were the dominant species among the gymnosperms. The number of bryophytes ranged from 0% to 2.08%, with an average of 0.45%; they were sporadically distributed in various coal seams (Table 1).

## 5. Discussion

### 5.1. Characteristics of Paleovegetation

The spore and pollen vegetation types were classified into coniferous trees, evergreen broad-leaved trees, deciduous broad-leaved trees, shrubs, and herbs [38,39]. In the present study area, the paleovegetation mainly comprised herbs, shrubs, broad-leaved trees, and coniferous trees. The herbs included *Fontinalaceae*, *Lycopodiaceae–Selaginellaceae*, *Hymenophyllaceae*, and *Ophioglossaceae*; the shrubs included *Osmundaceae*, *Cyatheaceae*, *Dipteridaceae*, *Sphenopsida*, *Gleicheniaceae*, *Lygodiaceae*, and *Polypodiaceae*; the broad-leaved trees included *Dicksoniaceae* and *Cycadaceae*; the coniferous trees included *Pteridospermae*, *Araucariaceae*, *Podocarpaceae*, *Cupressaceae*, *Cheirolepidiaceae*, and Conifers (Table 2).

According to the relationship between modern spores and pollen and the vegetational cover, some pollen, such as that from *Pinaceae* and *Cupressaceae* plants, was transported over long distances [40,41]. Although some spores and pollen were the result of long-distance transportation, the data on the spores and pollen can still be used to reconstruct the types of paleovegetation in the extra-thick coal seams from the Aalenian Stage in the ECDBs [42,43]. Therefore, the vertical succession of paleovegetation in the Aalenian Stage in the ECDBs was divided into three groups (Figure 3 and Figure 4; [33]).

Shrubs (20.62–45.72%) and broad-leaved trees (8.57–21.48%) were the dominant paleovegetation in Group A, and herbs (0–5.95%) and coniferous trees (2.42–14.38%) appeared less frequently at this stage. Ferns were at their peak, and gymnosperms were not predominant. The shrubs were mainly of the *Osmundaceae* (Baculatisporites) family, and the broad-leaved trees were mainly of the *Cyatheaceae* (Deltoidospora) and *Cycadaceae* (Cycadopites–Chasmatosporites) families [44,45,46].

The abundance of paleovegetation in Group B was higher than that in Group A due to the increased variety of gymnosperms. Shrubs (26.50–47.95%) remained the dominant paleovegetation, and broad-leaved trees (7.83–21.05%) decreased, while herb plants (5.79–18.88%) and coniferous trees (6.98–25.95%) increased. The shrubs were mainly from the *Osmundaceae* (Baculatisporides and Punctisporites) family, followed by the *Polypodiaceae* (Laevigatosporites) and *Dipteridaceae* (Dictyophyllidities) families. The number of ferns and broad-leaved trees decreased, while the changes in the *Cycadaceae* were not significant but still dominated by these two plants. The additional herbs were mainly from the *Lycopodiaceae–Selaginellaceae* (Aratrisporites, Neoraistrickia,) and *Hymenophyllaceae* (Lophotrilets) families, while the additional coniferous trees were mainly from the Conifers (Podocarpidites, Classopollis, Pristinuspollenites, Rugubivesiculites, and Cerebropollenites) family (Table 1).

In Group C, the proportion of shrubs (17.39–30.00%) decreased, while herbs (0–26.83%), broad-leaved trees (16.66–41.66%), and coniferous trees (8.32–40.00%) all increased. The number of gymnosperms increased sharply, while the number of ferns decreased sharply. At this point, the content of ferns was equivalent to that of gymnosperms. The shrubs were mainly from the *Osmundaceae* (Osmundachidites, Baculatiporites) and *Polypodiaceae* (Polypodisporites, Laevigatosporites) families, followed by the *Sphenopsida* (Calamospora). The herbs were still mainly from the *Lycopodiaceae–Selaginellaceae* (Densonisporites, Neoraistrickia) family, followed by the *Hymenophyllaceae* (Lophotrilets) and *Ophioglossaceae* (Undulatosporites) families. The broad-leaved trees were mainly from the *Cycadaceae* (Cycadopies–Chasmatosporites) family, with a significant decrease in the number of *Cyatheaceae* but a significant increase in the *Dicksoniaceae* (Cibotiumspora) family. The coniferous trees were still mainly composed of Conifers (Pinuspollenites, Cedripites, Pristinuspollenites, Quadraeculina, and Cerebrollenites). The abundance of pine and cypress plants reached its peak here (Table 1).

Compared with the paleovegetation in Yaojie of Gansu and the Dongsheng District of Erdos in Inner Mongolia, the number of *Cyatheaceae* plants in the study area was low, whereas that of *Osmundaceae* and Cladosporidae plants was high [36,37]. The number of gymnosperms from the extra-thick coal in the ECDBs was lower at the bottom of the coal seam but higher at the top. However, the number of gymnosperms in Yaojie was lower in the middle layer, which was different from that observed in the ECDBs. Additionally, the number of cycads was also higher than that in Yaojie and Dongsheng, whereas *Pinaceae* plants were dominant in Yaojie and Dongsheng [36,37]. Ferns developed in the ECDBs, but gymnosperms were more abundant in Yaojie and Dongsheng. Although two-bursa coniferous *Pinaceae* and broad-leaved Gymnospermae plants were developed in all three areas, *Pteridospermae* and *Podocarpaceae* plants developed in Yaojie and Dongsheng. The development of *Cupressaceae* plants in the Middle Jurassic period was significantly different from that in the ECDBs and Dongsheng (Table 1).

In summary, the paleovegetation in the ECDBs mainly included herbs of *Lycopodiaceae–Selaginellaceae* plants, shrubs of *Osmundaceae* and *Polypodiaceae* plants, broad-leaved trees of *Cyatheaceae* and *Cycadaceae* plants, and coniferous trees of pine and cypress plants. From the bottom to the top, the number of herbs and coniferous trees showed a continuous increase.

### 5.2. Characteristics of Paleoclimate

The spores and pollen in the dry–humid zone were classified into xeric, mesophytic, humidogenic, aquatic, and paludose plants [47,48,49]. By analyzing the impact of dryness and wetness, environmental changes were inferred, providing theoretical support for paleoclimate restoration. The spores and pollen in the climate zone were classified into tropical, subtropical, temperate, warmzone–subtropical, and tropical–temperate plants [47]. In this study, we divided the dry–humid zone into four categories: xeric, mesophytic, humidogenic, and aquatic. The proportions of temperate and subtropical plants in the research area were relatively low, so the area was classified as subtropical–temperate. During the statistical analysis, it was found that the aquatic plants included *Fontinalaceae*; the humidogenic plants included *Hymenophyllaceae*, *Sphenopsida*, *Osmundaceae*, *Cyatheaceae*, *Dicksoniaceae*, *Dipteridaceae*, *Gleicheniaceae*, *Lygodiaceae*, *Ophioglossaceae*, *Polypodiaceae*, *Podocarpaceae*, and *Cupressaceae*; the mesophytes included *Lycopodiaceae–Selagioellaceae*, *Cycadaceae*, and *Pineaceae*; and the xerophytes included *Pteridospermae*, *Araucariaceae*, and *Cheirolepidiaceae* [35,36]. In this study, the vegetation types, dry–humid zones, and climate zones were selected as the criteria to explore the features of the paleoclimate during the coal-bearing period of the extra-thick coal seams in the Aalenian Stage of the ECDBs (Table 2).

**Figure 7:**
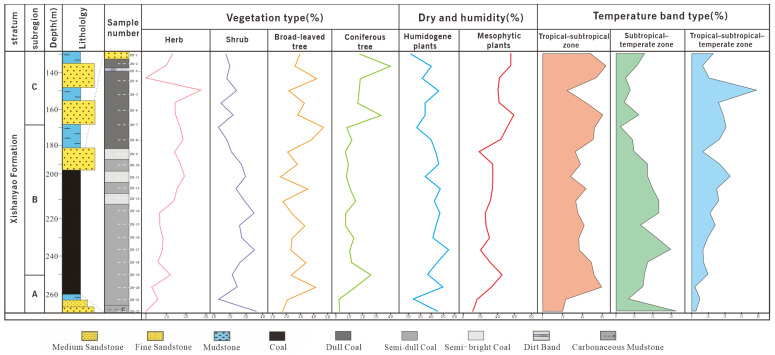
Vegetation, dry–humid zone, and climate trend diagram of the Aalenian Stage in the ECDBs.

Due to the relatively single trend of aquatic and xeric plants, which only appeared in some layers, we only made trend charts for humidogenic and mesophytic plants (Figure 7). We found that humidogenic plants showed an overall downward trend from the bottom to the top, while mesophytic plants showed an overall upward trend. Humidogenic plants ranged from 24.25% to 50.01% in Group A, from 34.44% to 55.09% in Group B, and from 21.74% to 46.34% in Group C. They were at their peak in ZS-4, ZS-10, ZS-12, ZS-14, ZS-17, and ZS-20, indicating that plants that prefer moisture, such as plants of the *Osmundaceae*, *Cyatheaceae*, *Dicksoniaceae*, and *Polypodiaceae* families, grew vigorously during this stage. Mesophytes ranged from 11.43% to 33.34% in Group A, from 18.62% to 45.91% in Group B, and from 41.47% to 60.34% in Group C. In Groups C and B, the peak of mesophytes at ZS 19 indicates that plants such as *Pinaceae* and *Lycopodiaceae–Selaginellaceae* grew vigorously.

Modern Filicinae plants are mainly distributed in tropical–subtropical zones. Deltoidospora and Cyathidites plants, belonging to the *Cyatheaceae* family, are broad-leaved plants living in tropical and subtropical humid areas [50,51]. *Filicinae* plants also include fossils of *Dipteridaceae* plants, which originated in the Late Triassic period. *Dipteridaceae* plants were found in the Late Jurassic period and were mainly distributed in the humid zone, with some distribution in the fluctuating tropical and subtropical zones during the Mesozoic period [52]. *Lycopodiaceae* plants now grow in tropical, subtropical, and temperate zones with humid climates; they are found in coniferous or coniferous broad-leaved mixed forests or among shrubs [53]. *Selaginellaceae* plants are mainly distributed in tropical, subtropical, and temperate zones and grow in wetlands, typically in the undergrowth or near streams [54]. *Cheirolepidiaceae* plants mainly grow in dry and hot climates, indicating a change in the paleoclimate from humid to dry conditions [52,55]. Podocarpidites plants are coniferous plants growing in semi-dry–semi-humid areas in tropical–subtropical zones. Cycadopites plants are broad-leaved plants growing in semi-dry–semi-humid areas in tropical–subtropical zones. Alisporites, Pinuspollenites, Erlianpollis, Piceaepollenites, Piceites, Pseudopicea, and Cedripites plants are coniferous plants growing in semi-dry–semi-humid areas in tropical–subtropical zones [56].

The relationships among the plant composition, dry–humid zones, and climate zone types show that the proportion of shrubs continuously decreased from the bottom to the top of the coal seam, peaked at the bottom of Group B, and then decreased in Group C. The number of coniferous plants increased continuously from Group A to Group C. In Group A, the number of coniferous plants was low. The number increased steadily in Group B and then displayed a sharp increase and peaked in Group C. The numbers of broad-leaved plants and herbs were insignificant for the whole area, but the number of herbs increased in Group B. The dry–humid zone showed an obvious change from the bottom to the top of the coal formation. A large number of humidogenic plants were found in Group A, but they decreased continuously. Conversely, the number of mesophytic plants increased. The number of xeric plants was lower in Group A; they began to appear in Group B and peaked in Group C. The growth habits of various plants indicate that the abundance of vegetation in the tropical–subtropical–temperate zone reached 30% and stayed around 15%. The subtropical–temperate plants mainly existed at the bottom of the coal seam, with an extremely low number at the top of the coal seam. The proportion of plants in the tropical–subtropical zone was higher than 40% and even reached up to 60% (Table 1 and Figure 7).

The paleoclimate in the study area experienced three stages according to the modern distribution of the main palynoflora, the dry–humid features, and the distribution of the climate zones. These three stages are warm–humid, humid–semi-humid, and semi-humid–semi-dry. The paleoclimate was mainly tropical–subtropical (Figure 7). A study on the paleoclimate of the Aalenian Stage in the middle-western Dzungaria Basin showed that the early Aalenian Stage presented a warm–humid climate. During the middle Aalenian Stage, the *Pinaceae* plants exhibited extremely vigorous growth, and the number of *Cheirolepidiaceae* plants increased, indicating that the paleoclimate became dry and hot [57,58]. This is consistent with the results of this study. During the Middle Jurassic period, the paleoclimate of the Yaojie area was mainly characterized by a transition between the semi-dry–semi-humid tropical–subtropical zone in the southeast and the dry tropical–subtropical zone in the southwest. The paleoclimate was not stable because of the seasonal transformation of the climate zone. Additionally, it changed from a warm–semi-humid state to a dry–humid state and, finally, to a cool–humid state. Large amounts of spores and pollen originated from the warm and wet plants in Donsheng, which implies a warm–humid climate with abundant plant growth. However, mesophytic *Pinaceae* plants also developed during the mid-Aalenian Stage [35,36].

During the Triassic–Jurassic Boundary period, the atmospheric CO_2_ levels increased rapidly. Additionally, intensive volcanic activity related to the Central Atlantic Magmatic Province also released large amounts of CO_2_. The resulting acute greenhouse effect led to global warming [59]. The rise in temperature increased the water vapor content in the upper troposphere. The amount of vapor in the upper troposphere was positively correlated with global lightning activity, leading to frequent forest fires [60,61,62,63,64]. Therefore, it can be deduced that the heating event in the Jurassic period may have been related to the occurrence of forest fires. In the early Aalenian Stage, the lake basin expanded continuously. This expansion of the sedimentary range led to differences in the altitude across the basin [65]. The developing hydrographic network from the Kelameili Mountains increased the water level in the lake. The increase in the lake surface area led to a humid environment, which encouraged the growth of shrubs and herbs. In the middle of the depositional stage, the expansion of the lake ceased, and the paleoclimate changed to the humid–semi-humid type [66]. During this time, the number of *Pinaceae* plants increased, whereas the number of ferns decreased. In the late depositional stage, differential uplift occurred in the basin, the lake bog shrank, and the paleoclimate became dry and hot. The hydrographic changes from the Kelameili Mountains decreased, leading to a continuous drop in the lake water level. The change in the paleoclimate led to the growth of coniferous plants [67]. *Cyatheaceae* and *Dicksoniaceae* plants were broad-leaved ferns of *Filicinae* that primarily grew in warm–humid zones. The decrease in the number of *Cyatheaceae* plants from the bottom to the top further validates the change in the paleoclimate from warm–humid to dry–hot [68,69,70]. *Cheirolepidiaceae* were seasonal xeric plants, including Classopollis, which mainly grew on slopes. These xeric plants were less abundant in Group C but increased in proportion in Group B, which further proves the change in the paleoclimate.

In conclusion, the study area experienced a heating event, which led to large-scale lake transgression and retreat during the depositional stage of the Aalenian Stage. The paleoclimate transitioned from warm–humid to tropical–subtropical to humid–semi-humid and, finally, to semi-humid–semi-dry.

### 5.3. Reconstruction of the Paleovegetation Community

Based on the previous findings, the paleovegetation community of the extra-thick coal seam of the Xishayao Formation in the ECDBs was reconstructed. Typically, the nearshore wetland and lowland plant communities included in situ-buried plants, which were mainly coal-bearing plants. The seeds, spores, and pollen from the *Pinaceae* plants in the abrupt slope and highland zones were transported to the depositional areas by wind and water, resulting from allochthonous transportation [35,71,72].

#### 5.3.1. Nearshore Wetland Paleovegetation Community

**Figure 8:**
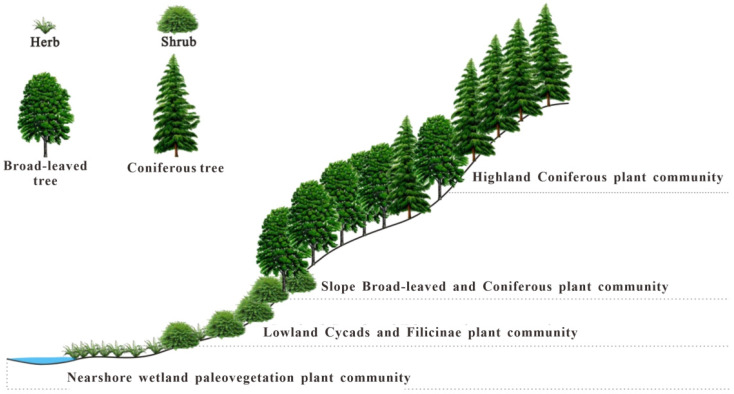
Patterns of paleovegetation community.

These plants were mainly distributed along the damp banks of lakes and rivers and in the undergrowth. The swampland and the low-lying humid areas were also favorable for the growth of these plants. These plants were mainly wetland plants with weak hydrodynamic conditions [35], enabling sporomorphs to be preserved in situ. The dominant species included *Sphenopsida*, *Equisetum*, and some *Pteridophyta* and *Musci* plants, such as Calamospora, Hymenophyllumsporites, Undulatisporites, Cicatricosisporites, Klukisporites, and Sphagnumsporites. This plant community was simple and had low herbaceous growth (Figure 8).

#### 5.3.2. Lowland Cycad and Filicinae Plant Community

This plant community was mainly found in areas with slight undulation, damp soil, and good permeability. It comprised Filicopsida and Cycadopsida plants, including Osmundacidites, Cyclogranisporites, Baculatisporites, Punctatisporites, Todisporites, Aratrisporites, Lycopodiumsporites, Neoraistrickia, Cyathidites, Deltoidospora, Cibotiumspora, Dictyophyllidites, Concavisporites, Gleicheniidites, Polypodiisporites, Laevigatosporites, Cycadopites, Monosulcites, and Eucommiidites. These plants were shrubs and broad-leaved trees, with Bennettitinae being one of them. The species diversity of *Filicinae* plants was high, so they were the dominant species in this community. The appearance and structure of this community included prolific *Filicinae* plants, low herbaceous *Filicinae* plants, shrubs, and low xylophytes. Overall, this plant community comprised shrubs and ferns under broad-leaved trees (Figure 8).

#### 5.3.3. Slope Broad-Leaved and Coniferous Plant Mixed Community

This plant community was distributed around mountainous slopes or hills inside the basin. With steeper slopes and good permeability, the dominant plants included coniferous and broad-leaved mesophytes, comprising both trees and shrubs. Herbaceous *Filicinae* plants grew under the vegetation and along the damp hillside edge. *Cycads*, *Cupressaceae*, and *Pinaceae* plants were dominant species, including Podocarpidites, Pinuspollenites, Laricoidites, Rugubivesiculites, Pseudopicea, Quadraeculina, Cerebropollenites, and Perinopollenites. In this community, coniferous *Pinaceae* plants occupied the high hillside slopes, the middle hillside slopes had mixed coniferous and broad-leaved trees, and the lower hillside slopes mainly had broad-leaved trees and shrubs. Meanwhile, ferns still grew in the wet belt between the forest and valley slopes (Figure 8).

#### 5.3.4. Highland Coniferous Tree Community

This plant community grew at the top of the mountains or in high areas inside the basin. The dominant species were *Cupressaceae* and *Pinaceae* plants, including Piceaepollenites, Cedripites, and Callialasporites. Xeric plants from *Cheirolopidiaceae* and *Pteridospermae*, such as Caytonipollenites and Alisporites, also grew here. This plant community mainly grew in relatively high-altitude areas with low temperatures and relatively dry soil [67]. Additionally, the coniferous vegetation structure was simple, with fewer humidogenic herbs and shrubs (Figure 8).

### 5.4. Formation and Thinning of Extra-Thick Coal Seams

**Figure 9:**
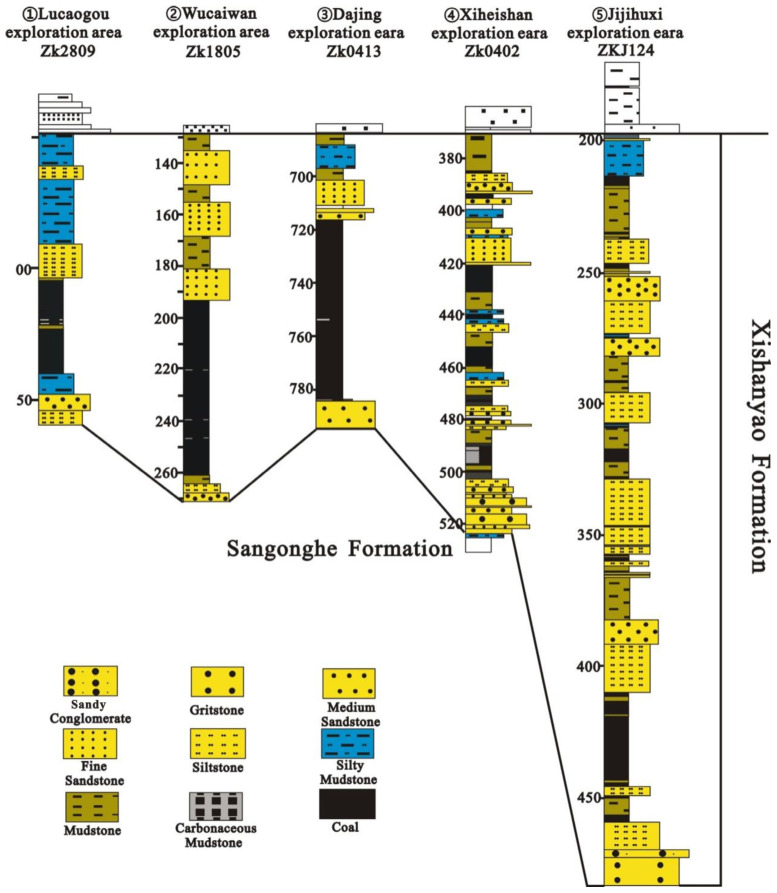
Trend chart of coal seam thickness changes in the ECDBs (modified from [30]).

The accumulation and formation of peat swamps are determined by two basic conditions: (1) prosperous plant communities in the swamps and (2) sufficient space and good preservation conditions for the accumulation of peat layers [73,74]. Due to the ferns flourishing in the study area, the growing environment comprised mixed swamps, which facilitated rapid accumulation and preservation. The corresponding paleovegetation communities included the nearshore wetland paleovegetation community, the lowland cycads, and the *Filicinae* plant community. However, the slope broad-leaved and coniferous plant mixed community and highland coniferous tree community included mainly gymnosperms and broad-leaved ferns. The plant remains from these communities were transported over long distances with their sources. Therefore, it is presumed that the plant remains on the slopes and plateaus carried by water are located in the middle and south of the ECDBs. The scale of transgressions plays a significant role in the accumulation of peat swamps. Coal with economic value can only form when the rate of increase in accommodation space caused by transgression approaches or slightly exceeds the rate of peat accumulation [75]. If the transgression rate is high enough to submerge the peat swamps, plant growth is inhibited, resulting in the formation of shallow lake sediments [76]. Similarly, peat swamps are oxidized in a regressive environment. Neither condition is conducive to the formation or development of peat swamps. Due to a warming event in the Middle Jurassic period, the coal seam in the Aalenian Stage underwent large-scale lake transgression and regression during deposition, accompanied by the transfer of the coal-accumulating center. During the early stage of the expansion of the lake basin from the southeast to the northeast, the coal-accumulating center migrated from the east to the west and southwest of the ECDBs. In the later stage of lake basin shrinkage, the coal-accumulating center migrated to the middle and southeast of the Dajing mining district [77]. These depositional features determined the formation of the extra-thick coal seams in the Wucaiwan and Dajing mining districts northwest of the ECDBs. The periodic small-scale transgressive–regressive cycles resulted in a large number of thin coal seams in the east and southeast of the ECDBs (Figure 1 and Figure 9).

## 6. Conclusions

The conclusions of this study are as follows:

(1) The types of spores and pollen belong to forty-five genera, including twenty-three fern, twenty gymnosperm, and two bryophyte genera, in the extra-thick coal seams (Aalenian Stage) of the ECDBs, with ferns as the main spore and pollen fossils, followed by gymnosperms. The paleovegetation types in the study area mainly included *Lycopodiaceae–Selaginellaceae* herbs, *Osmundaceae* and *Polypodiaceae* shrubs, and *Cyatheaceae*, *Cycadaceae*, and *Pinaceae* coniferous broad-leaved trees. From the bottom to the top, the number of ferns increased first and then decreased, and the number of gymnosperms continued to increase. The number of herbs and *Pinaceae* plants showed a continuous increase.
(2) During the sedimentary period, the paleoclimate of the Aalenian Stage in the ECDBs changed from warm–humid to humid–semi-humid and, finally, to semi-humid–semi-dry, all within the tropical–subtropical zone.
(3) The study area was divided into four paleovegetation communities: nearshore wetland paleovegetation community, lowland cycad and Filicinae plant community, mixed community of slope broad-leaved and coniferous trees, and highland coniferous tree community. The nearshore wetland paleovegetation community and lowland cycad and Filicinae plant community were the main coal-forming plant communities in the study area.
(4) According to the analysis of the characteristics of paleovegetation types and the paleoclimate succession model, a warming event occurred in the Dzungaria Basin during the Middle Jurassic period. This event resulted in large-scale lake transgression and regression in the basin, leading to a shift in the coal-accumulating center from the west and southwest to the central part of the ECDBs.

## Figures and Tables

**Table 1:** Statistical analysis of spore and pollen fossils in extra-thick coal seams of the Aalenian Stage in the ECDBs (%).

Parent Plant	Group A	Mean Value	Group B	Mean Value	Group C	Mean Value	Yaojie Formation, Gansu	Dongsheng, Neimenggu
*Fontinalaceae*	0~1.11	0.37	0~2.22	0.51	0~2.08	0.45	/	/
*Lycopodiaceae* *Selaginellaceae*	0~2.42	0.81	3.06~12.22	6.93	0~19.51	8.75	0~0.50	13.00~20.00
*Hymenophyllaceae*	0~2.30	0.77	0~6.02	2.97	0~4.88	2.63	/	/
*Sphenopsida*	0~4.02	1.54	0~3.62	1.48	0~4.17	0.98	/	/
*Osmundaceae*	8.48~34.29	18.85	4.80~27.55	16.97	2.44~13.04	7.39	0.50~2.40	5.00~13.00
*Cyatheaceae*	7.88~20.11	12.19	0~16.52	9.70	0~10.72	6.00	1.20~61.70	24.00~45.00
*Dicksoniaceae*	0~1.72	0.98	0~6.02	2.07	0~12.19	4.79	0.50~1.10	4.00~6.00
*Dipteridaceae*	0~1.73	0.78	0.87~9.30	3.01	0~2.44	0.73	0.40~2.00	11.00~21.00
*Gleicheniaceae*	0~0.61	0.20	0~0.83	0.07	0~6.67	1.11	/	0~13.00
*Lygodiaceae*	0~3.46	1.36	0~2.54	0.72	0~3.57	0.74	/	/
*Ophioglossaceae*	0~2.42	1.00	0~6.98	1.64	0~4.17	1.73	0.50~1.50	12.00~19.00
*Polypodiaceae*	1.72~2.86	2.13	1.65~7.78	4.67	3.33~19.51	7.41	0~1.10	/
*Pteridospermae*	0~0.58	0.19	0~2.18	0.53	0~4.16	1.33	1.60~8.00	4.00~15.00
*Cycadaceae*	8.57~20.12	13.40	6.96~24.1	14.96	9.76~37.49	22.36	0.40~25.60	18.00~23.00
*Araucariaceae*	/	/	0~1.05	0.16	0~2.44	0.58	0.50~0.90	4.00~7.00
*Podocarpaceae*	/	/	0~3.99	0.49	/	/	0.40~3.80	5.00~9.00
*Cupressaceae*	0~0.58	0.19	/	/	0~2.44	0.35	0.70~9.00	/
*Cheirolepidiaceae*	/	/	0~1.69	0.37	/	/	0.40~1.00	3.00~4.00
*Pinaceae*	2.42~6.33	3.87	0~8.70	2.21	0~14.29	5.72	1.50~9.70	39.00~77.00
*Pinaceae*, unknown classification	0~6.89	2.30	3.33~19.69	7.76	0~40	13.50	6.50~47.90	22.00~31.00

Yaojie Formation, Gansu, data from reference [36]; Dongsheng, Neimenggu, data from reference [37].

**Table 2:** Vegetation types, dry–humid zones, and climate zones in the Aalenian Stage in the ECDBs (modified from [37]).

Parent Plant	Vegetation Type	Dry–Humid Zone	Climate Zone
*Fontinalaceae*	Herb	Aquatic plant	Tropical–subtropical–temperate
*Lycopodiaceae*– *Selaginellaceae*	Herb	Mesophytic plant	Tropical–subtropical–temperate
*Hymenophyllaceae*	Herb	Humidogenic plant	Subtropical
*Sphenopsida*	Shrub	Humidogenic plant	Tropical–subtropical
*Osmundaceae*	Shrub	Humidogenic plant	Subtropical–temperate
*Cyatheaceae*	Shrub	Humidogenic plant	Tropical–subtropical
*Dicksoniaceae*	Broad-leaved tree	Humidogenic plant	Tropical
*Dipteridaceae*	Shrub	Humidogenic plant	Tropical–subtropical
*Gleicheniaceae*	Shrub	Humidogenic plant	Tropical–subtropical
*Lygodiaceae*	Shrub	Humidogenic plant	Tropical
*Ophioglossaceae*	Herb	Humidogenic plant	Tropical
*Polypodiaceae*	Shrub	Humidogenic plant	Tropical–subtropical–temperate
*Pteridospermae*	Coniferous tree	Xeric plant	Tropical–subtropical–temperate
*Cycadaceae*	Broad-leaved tree	Mesophytic plant	Tropical–subtropical
*Araucariaceae*	Coniferous tree	Xeric plant	Tropical
*Podocarpaceae*	Coniferous tree	Humidogenic plant	Tropical–subtropical
*Cupressaceae*	Coniferous tree	Humidogenic plant	Temperate
*Cheirolepidiaceae*	Coniferous tree	Xeric plant	Tropical–subtropical–temperate
*Pinaceae*	Coniferous tree	Mesophytic plant	Tropical–subtropical
*Pinaceae*, unknown classification	Coniferous tree	Mesophytic plant	Tropical–subtropical

## Data Availability

Data is contained within the article.
